# Understanding Ovarian Cancer: iTRAQ-Based Proteomics for Biomarker Discovery

**DOI:** 10.3390/ijms19082240

**Published:** 2018-07-31

**Authors:** Agata Swiatly, Agnieszka Horala, Jan Matysiak, Joanna Hajduk, Ewa Nowak-Markwitz, Zenon J. Kokot

**Affiliations:** 1 Department of Inorganic and Analytical Chemistry, Poznan University of Medical Sciences, ul. Grunwaldzka 6, 60-780 Poznań, Poland; agataswiatly@gmail.com (A.S.); jmatysiak@ump.edu.pl (J.M.); jo.hajduk@gmail.com (J.H.); 2 Gynecologic Oncology Department, Poznan University of Medical Sciences, ul. Polna 33, 60-535 Poznań, Poland; agnieszka0lemanska@gmail.com (A.H.); ewamarkwitz@poczta.fm (E.N.-M.)

**Keywords:** proteomics, ovarian cancer, isobaric tags, carcinogenesis, MALDI-TOF MS

## Abstract

Despite many years of studies, ovarian cancer remains one of the top ten cancers worldwide. Its high mortality rate is mainly due to lack of sufficient diagnostic methods. For this reason, our research focused on the identification of blood markers whose appearance would precede the clinical manifestation of the disease. ITRAQ-tagging (isobaric Tags for Relative and Absolute Quantification) coupled with mass spectrometry technology was applied. Three groups of samples derived from patients with: ovarian cancer, benign ovarian tumor, and healthy controls, were examined. Mass spectrometry analysis allowed for highlighting the dysregulation of several proteins associated with ovarian cancer. Further validation of the obtained results indicated that five proteins (Serotransferrin, Amyloid A1, Hemopexin, C-reactive protein, Albumin) were differentially expressed in ovarian cancer group. Interestingly, the addition of Albumin, Serotransferrin, and Amyloid A1 to CA125 (cancer antigen 125) and HE4 (human epididymis protein4) improved the diagnostic performance of the model discriminating between benign and malignant tumors. Identified proteins shed light on the molecular signaling pathways that are associated with ovarian cancer development and should be further investigated in future studies. Our findings indicate five proteins with a strong potential to use in a multimarker test for screening and detection of ovarian cancer.

## 1. Introduction

Despite intensive research ongoing for decades, no effective ovarian cancer (OC) diagnostic methods are available. With an incidence rate of 6.1 and age-standardized mortality rate of 3.7, OC remains among the ten top cancers worldwide. The rates are even higher in Europe (9.9 and 5.4, respectively) [1]. Due to lack of early symptoms, OC is usually detected in advanced stages. The advances in early diagnosis could significantly improve the treatment outcome as the five-year survival rate for the disease detected in stage I (according to classification by International Federation of Gynaecology and Obstetrics—FIGO) reaches 90% and drops below 30% for stages III-IV, according to FIGO [2].

Diagnostic methods that are currently used in practice encompass gynaecological transvaginal examination, ultrasound imaging and biomarkers, such as cancer antigen 125 (CA125) and human epididymis protein 4 (HE4). Various indexes have been constructed using the combinations of clinical data (such as age), specific ultrasound features and aforementioned protein levels are used for risk assessment, e.g., Risk of Malignancy Index (RMI) that is calculated on the basis of CA125 serum level, the menopausal status of the patient, and five ultrasound features. However, the common limitation of those methods is the fact that they can be applied in clinical setting for treatment planning only after an ovarian tumor is diagnosed by imaging methods and not for early diagnosis. For this reason, our research focuses on searching for the blood markers of OC. Their presence could precede the clinical manifestation of the disease. We have previously published papers that have contributed to the widening of knowledge on OC pathogenesis and pathological processes that are associated with its growth identifying metabolic pathways and potential OC biomarkers [3,4,5]. The identification of specific disease indicator(s) in blood could enable the elaboration of a new non-invasive diagnostic method and/or improve the pre-operative differential diagnosis of ovarian tumors, and thus lead to a reduction of unnecessary surgical procedures. Therefore, this study aimed to identify proteins that are differently expressed during OC development. Our findings may provide valuable insight into the complex molecular mechanism of carcinogenesis.

**Figure 1:**
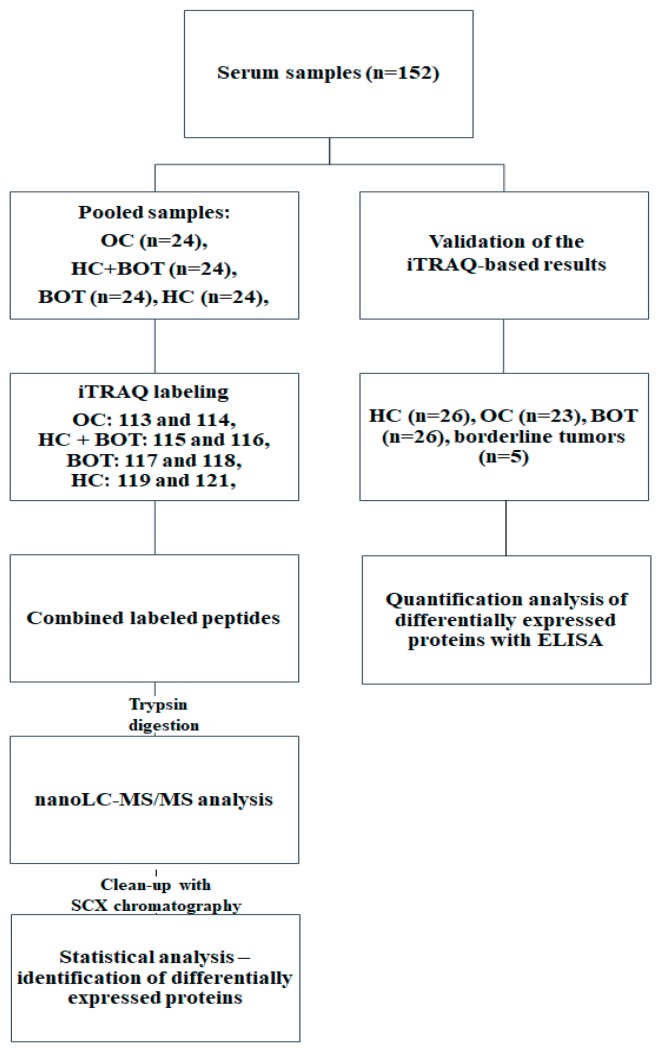
Schematic workflow of the study. Three groups of samples were analyzed: OC ovarian cancer; BOT benign ovarian tumor; HC healthy controls.

Recently, quantitative proteomics has emerged as a powerful technology for searching for cancer-specific proteins. Isotopic labelling, especially methods, like SILAC (stable isotope labelling by amino acids in cell culture), ICAT (Isotope-coded affinity tag) or iTRAQ (isobaric Tags for Relative and Absolute Quantification), have become standard tools for identifying differences in protein expression. In the field of OC research, isobaric tags were used e.g., for proteomic characterization of OC cells [6], differentiation between benign and malignant OC tissues [7], treatment monitoring [8], or the identification of biomarkers useful in screening [9]. In this study, we decided to use iTRAQ technology combined with matrix-assisted laser desorption/ionization time-of-flight mass spectrometry (MALDI-TOF MS), which proved to be an efficient significant method for biomarker discovery. Serum proteins levels from three groups of samples: OC, benign ovarian tumors (BOT), and healthy controls (HC), were quantified. The study workflow is shown in Figure 1. To the best of our knowledge, only a few studies encompassing all three groups of patients were published so far. Subsequently, an external validation set of samples was tested with ELISA (enzyme-linked immunosorbent assay) method to assess the obtained results.

## 2. Results

**Figure 2:**
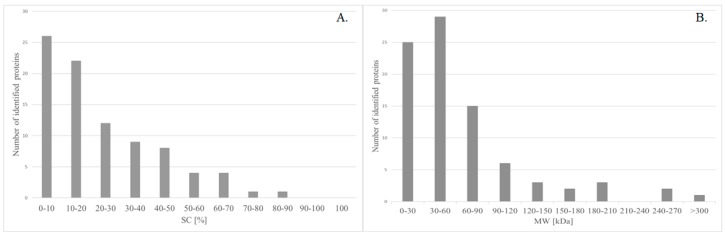
Distribution of all identified proteins in mass spectrometry analysis based on (**A**) Sequence Coverage (**B**) Molecular Weight.

3217 unique peaks were identified which corresponded to 114 proteins. However, in order to obtain reliable results, only proteins identified with at least two unique peaks and Mascot Score > 80 were further analyzed. The average sequences coverage (SC) of peptides was 23.97%, and the distribution of proteins that are based on SC is presented in the Figure 2A. 33.72% of the identified proteins had the molecular weight (MW) between 30–60 kDa, 29.07% between 0–30 kDa, 17.44% between 60–90 kDa, and the rest was larger than 120 kDa (Figure 2B). These results show that a variety of proteins with different MW were identified.

ITRAQ ratios for four different comparisons were calculated: OC vs. HC, OC vs. BOT, OC vs. HC + BOT, BOT vs. HC. The observed differences in protein expression, according to ratios, are presented in Table 1. Significant differences in the serum levels of 11 proteins were observed. In general, C-reactive protein (CRP), Fibrinogen alpha chain (FGA), Apolipoprotein C-II (APOC2), and Serum amyloid A1 protein (SAA1) were up-regulated and Alpha-1-acid glycoprotein 1 (ORM1), Alpha-1-antitrypsin (SERPINA1), Hemopexin (HPX), Serotransferrin (TF), and Serum albumin (ALB) were down-regulated in OC when compared to other groups. While HPX was up-regulated, other proteins (SERPINA1, APOC2, Apolipoprotein C-IV-APOC4, and Protein Z-dependent protease inhibitor—SERPINA10) were down-regulated in BOT compared with HC. CRP, FGA and SAA1 significantly differed in all comparisons with OC, but no difference was observed between BOT and HC. Therefore, they were expected to identify OC patients better than other proteins. Protein APOC2 was differently expressed in BOT, which suggests its role as a BOT marker and it could be useful in differential diagnosis of ovarian tumors. Raw data obtained in the ProteinScape software are shown in the Table S1. Discrepancies in the coefficient of variation (CV) values were observed. The protein variability in reporter ion signals may be associated with two separate repetitions of the experiment.

**Figure 3:**
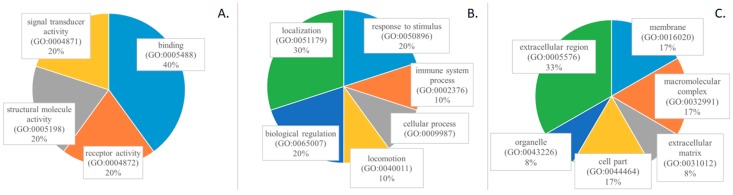
Gene ontology analysis of the most discriminative 11 proteins identified in iTRAQ analysis based on PANTHER database, including: (**A**) molecular function; (**B**) biological process; and, (**C**) cell component.

Gene ontology analysis was conducted using protein annotation through evolutionary relationship (PANTHER) [10] for all identified proteins and also separately only for differently abundant proteins. According to database analysis, most of the identified proteins are associated with catalytic activity, binding and transporter activity, contribute to different biological processes, and are present in the membrane, extracellular region, and macromolecular complex. Proteins that are selected as potential OC indicators have different molecular functions: binding, receptor activity, signal transducer activity, and structural molecule activity (Figure 3). They participate in biological processes, like biological regulation, cellular processes, immune system processes, localization, locomotion, and response to a stimulus. They are a component of cell part, extracellular matrix, and region, macromolecular complex, membrane, and organelle.

**Figure 4:**
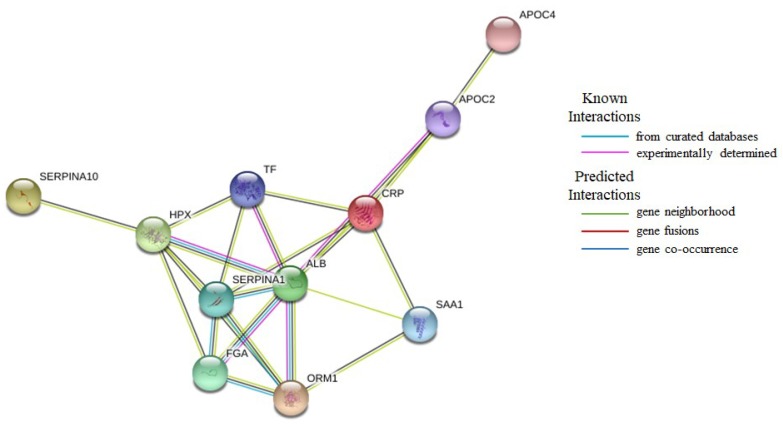
Interaction network of proteins identified as differentially expressed using iTRAQ analysis (source: https://string-db.org): C-reactive protein (CRP), Fibrinogen alpha chain (FGA), Apolipoprotein C-II (APOC2), Apolipoprotein IV (APOC4), Serum amyloid A1 protein (SAA1), Alpha-1-acid glycoprotein 1 (ORM1), Alpha-1-antitrypsin (SERPINA1), Hemopexin (HPX), Serotransferrin (TF), Protein Z-dependent protease inhibitor (SERPINA10) and Serum albumin (ALB).

STRING portal was used to analyze protein-protein interaction network (Figure 4). Network analysis allowed for the identification of various possible interactions. Several proteins are linked with even more than four different interactors. The pivotal roles in the obtained network play: SERPINA1, CRP, FGA, HPX, ORM1, TF, and ALB.

Validation of 10 differently abundant proteins that were identified by iTRAQ-based mass spectrometry procedure was conducted using commercially available ELISA kits. Since ELISA kit for measurement of serum concentrations of fibrinogen alpha chain is not commercially available, we did not validate results that were derived from this protein. ELISA confirmed the up-regulation of CRP and SAA1 and down-regulation of TF, HPX, and ALB in OC when comparing with BOT and HC. None of the determined proteins was significantly different in BOT compared with HC. To determine the potential ability of selected proteins to discriminate the studied groups, ROC curves were calculated. Statistical results based on ELISA are presented in Table 2. Moreover, for comparison of OC and BOT, *p*-values and AUC were calculated for two proteins: CA125 and HE4, routinely measured in hospital.

The highest AUC in the comparisons OC vs. BOT + HC and OC vs. BOT was obtained by TF (0.851), followed closely by SAA1, while OC and BOTs were best discriminated by ALB (AUC = 0.875), SAA1 (AUC = 0.851), and TF (AUC = 0.858). High AUC values were also obtained by HPX (0.762–0.796) and CRP (0.761–0.793). Nevertheless, no new protein exceeded the AUC values of CA125 (AUC = 0.965) and HE4 (AUC = 1.0) in differential diagnosis of ovarian tumors (OC vs. BOT). The significance of APOC2, APOC4, ORM1, SERPINA1, and SERPINA10 in differential diagnosis of ovarian tumors was not confirmed. No protein proved effective in differentiating between BOT and HC, which may suggest that the presence of BOT is not associated with extensive metabolic dysregulation.

In the next step, multivariate ROC curves were generated for the most discriminative proteins. For OC vs HC + BOT, panel of CRP, SAA1, TF, HPX, and ALB was characterized by AUC equal 0.936. The same model obtained an AUC = 0.903 in the comparison OC vs. HC. AUC of 0.972 was achieved by a model combining CRP, SAA1, TF, HPX, and ALB for distinguishing between OC and BOT, while AUC of 0.983 was obtained for the combination of CA125 and HE4. The addition of ALB, SAA1, and TF to a model containing CA125 and HE4 further increased AUC from 0.983 to 0.987. Furthermore, the potential usefulness of quantified proteins in discriminating borderline tumors from BOT and OC was tested (Table S4). The concentrations of SAA1, TF, HPX, and ALB significantly differed in borderline tumors and OC. Interestingly, APOC2, SERPINA1, and SERPINA10 obtained the AUC of 0.782, 0.739, and 0.748, respectively. Only CRP was upregulated in the borderline tumor group when compared with BOT.

Subsequently, OC group was divided, according to FIGO stages: stages I + II and stages III + IV. Results from ANOVA analysis indicated that CRP is significantly upregulated, while ALB concentration is significantly downregulated in stages III + IV. Not surprisingly, all five proteins: CRP, HPX, TF, ALB, and SAA1, were different expressed in the late stages of OC (III + IV) when comparing with BOT and HC. ALB, SAA1 and TF were also dysregulated in the early stages (I + II). Detailed results are presented in Table S5. Due to small set of samples representing each FIGO stage, further analyses are needed to confirm those results.

## 3. Discussion

Isobaric tags have already been used to investigate many aspects of OC. Since chemo-resistance may occur as one of the major problem during OC treatment, iTRAQ technology was used to identify proteomic signatures that are associated with its incidence [11,12,13]. Moreover, several significant studies aiming at the discovery of crucial proteins for OC development were based on the iTRAQ-analysis of the tissues [14,15], tumor fluids [16,17], and serum [9,18]. In this study, due to inexpensive and minimally invasive collection procedure, serum samples were used. Furthermore, CA125 and HE4—clinically used OC markers—were measured in the serum of patients with ovarian tumors (OC and BOT groups) as part of the standard hospital procedure. We decided to compare three groups of samples: epithelial OC, HC with no changes in the ovaries, and, what should be emphasized, BOT group. So far, the only reliable method of distinguishing between benign and malignant ovarian tumor requires histological analysis of the resected tissue. Thus, the introduction of novel non-invasive differentiation methods may contribute to a reduction in unnecessary surgical procedures and optimal qualification for treatment [19].

The performed iTRAQ-based analyses identified several proteins, which have already been suggested as potential OC biomarkers (TF, SAA1, CRP, ALB) [20,21,22,23]. These findings confirmed that isobaric tags are a proper method for identifying differences in serum protein expression. Gene ontology analysis demonstrated that the most discriminative proteins differ in molecular functions, involvement in biological processes, and interaction with cell components. On the other hand, STRING analysis provided information on correlations and interactions between the studied proteins. ELISA measurements of the proteins selected in the iTRAQ analysis was an important stage of our research. External validation conducted on a heterogeneous external dataset provides the highest irrefutable evidence of the reliability of the obtained results [24]. ELISA analysis confirmed that TF, SAA1, HPX, CRP, and ALB are strongly correlated with OC, when comparing with both, BOT and HC. Interestingly, TF, SAA1, HPX, and ALB also distinguished OC from borderline tumors. TF, SAA1, and ALB were also significantly dysregulated in early FIGO stages of OC (I+II) when comparing with BOT and HC, which may be relevant finding for further studies. However, the number of samples from patients with borderline tumor and early stages of OC was too small and the analysis should be continued on a larger group of patients (Tables S3 and S5). Nevertheless, no new protein exceeded the discriminative ability of CA125 and HE4 in the differential diagnosis of ovarian tumors (OC vs. BOT). For technical reasons, CA125 and HE4 were not identified in our MS analysis. CA125 consists of different forms of glycoproteins with molecular weight ranging from 110 kDa to over 2000 kDa, and it is commonly identified and quantified while using immunoassays. However, due to inconsistencies in molecular structure, there are only few papers describing MS-based identification of CA125 [25]. Similarly, HE4 is a glycoprotein mainly analyzed using immunoassays. However, HE4 is characterized by lower mass than CA125 (about 20 kDa) and its identification in serum samples using MS method requires special protocols, like immunoprecipitation [26]. Moreover our research was not focused specifically on glycoproteomics.

TF, SAA1, CRP, ALB, and HPX were identified as the most discriminative molecules for OC among the investigated proteins. Thus, we decided to discuss their role in molecular pathways associated with carcinogenesis. The main function of TF is to bind and deliver iron in blood from sites of absorption and red blood cell degradation to all cells and tissues that are involved in erythropoiesis and proliferation. Iron is necessary for cell proliferation and its deficiencies leads to apoptosis. In normal, healthy, human tissue, the TF receptors (TfRs 1 and TfRs 2), are expressed at low levels. However, on highly proliferative cells, like cancer cells, receptors, especially TfRs 1, are overexpressed and iron consumption is at a higher rate [27]. Thus, many studies suggested the using of TfRs in targeted therapies, for the delivery of medication directly to the tumor [28]. However, carcinogenesis leads to decrease of levels of TF in patients’ blood. Inflammatory cytokines, which are released during tumor development, reduce iron availability by stimulating hepcidin, while hepcidin blocks iron input into the circulating [29]. Iron deficiency is reflected by the level of TF. TF is already used in clinical practice as a part of a multi-marker serum test (OVA1) approved by the FDA for OC risk estimation. OVA1 test, apart from TF, is based on of CA125, transthyretin, apolipoprotein A-1, and β2-microglobulin [20]. Under-expression of TF in both, serum and cancer tissue samples, was also proved by a label-free LC-MS experiment [30]. Another MS-based proteomic analysis using data-independent acquisition and parallel reaction monitoring confirmed that TF is a reliable OC biomarker [31]. Moreover, the concentration of TF was significantly altered during the development of serous OC in a rat model [32].

The expression of acute phase proteins—SAA is mainly regulated by pro-inflammatory cytokines and their synthesis increases during acute and chronic inflammatory diseases. There are four different isoforms of SAA: SAA1, SAA2, SAA3, and SAA4. SAA1, and SAA2 are produced during inflammatory processes, and SAA4 is constitutively expressed in the liver [33]. SAA levels reach the highest point on the third day after the typical acute event, and about seventh day after the acute event, SAA rate return to baseline. Therefore, it can be suggested that one of the SAA roles is to maintain homeostasis. However, cancer cell lines and other extrahepatic tissues, like cancer metastases, are also able to stimulate SAA synthesis. During chronic inflammation, SAA, and other pro-inflammatory proteins levels are extensively increased, and they cannot be balanced by any anti-inflammatory molecules. Moreover, the activation of inflammatory cells by the cancer cells may result in an intensification of cancer progression [34]. Therefore, SAA was proposed as a potential non-specific prognostic biomarker of several malignancies and of cancer progression [35,36]. The expression of SAA gradually increased during progression from BOT and borderline tumors to OC [37]. SAA1 was also identified by SELDI-TOF MS, and further validated by immunoassay, as a potential biomarker of OC [21,38]. Moreover, up-regulation of SAA1 was observed in OC patients by Rauniyar et al. [31]. Finally, the study of ovarian cyst fluid allowed to select SAA4 as a possible ovarian-tumor-specific biomarker [16].

CRP is another acute phase protein identified in our study. It is routinely used in clinical practice as a marker of inflammation that is produced by hepatocytes and regulated by pro-inflammatory cytokines (mostly interleukin-6). The relationship between high concentrations of CRP and the risk of different cancers was demonstrated by several studies [39,40]. There are two hypotheses that are explaining the association between CRP and cancer. The first one assumes that a high CRP level is a result of underlying. The second one is reverse—it suggests that CRP and inflammation processes contribute to cancer development and progression. The initiation of mutations of tumor-suppressor genes, as well as post-translational modifications of proteins responsible for DNA repair and regulation of apoptosis, occur due to oxidative damage that is caused by inflammation [41]. The correlation between CRP and OC risk was also explored with one-time measurement [42] and longitudinal study [22]. It was proved that elevated CRP concentrations are associated with a higher risk of OC [22,43]. Therefore, it could be suggested that inflammation processes precede and/or promote OC development and acute phase proteins, including SAA1 and CRP, could be of the main markers of ovarian carcinogenesis. Inflammatory pathways might have a causal role in the inhibition of the cancer cells apoptosis and promotion of angiogenesis, vascular permeability, and proliferation [41].

Serum albumin—a protein down-regulated in OC group—is one of the major components of the human blood and it is characterized by a good binding capacity for many substances, e.g., drugs, hormones, zinc, calcium, sodium, potassium, fatty acids, or bilirubin. Albumin mainly participates in transport of the substances in blood and regulation of intravascular oncotic pressure [44]. The amount of albumin reflects a balance between processes of syntesis in the liver and peripheral degradation. Synthesis of albumin by hepatocytes may be suppressed by inflammation processes. During carcinogenesis pro-inflammatory cytokines, like interleukin-6, and growth factors are released, which stimulate liver production of acute phase proteins (SAA, CRP etc.,). Therefore, interleukin-6 can modulate synthesis of albumin in liver and decrease its level [45]. Interestingly, albumin and other macromolecules (>40 kDa) may be accumulated within the solid tumor interstitium, due to highly permeable vasculature and a lack of sufficient lymphatic drainage. Thus, this process is noted as the enhanced effect of permeation and retention. Moreover, it has been also suggested that albumin is catabolized in tumors [44]. Albumin has been considered as a prognostic marker in OC for a long time [46]. Low albumin level is associated with many malignancies, like colorectal cancer [47] or breast cancer [48]. The malignant disease can inhibit liver synthesis of albumin, which corresponds to its decreasing concentration during carcinogenesis. Albumin was proved to be a significant predictor of survival in OC and according to Asher et al. albumin concentrations below 25 g/L were associated with median survival of 4.8 months and above 35 g/L with a median survival of 43.2 months [23].

The last protein with significantly altered expression, according to ELISA and iTRAQ analysis is HPX. HPX is acute phase glycoprotein able to bind heme and transport it to the liver. Protection of the body from oxidative stress and damage is one of the main functions of this protein. Synthesis of HPX, like other inflammatory proteins (CRP, SAA), is modulated by cytokines. Surprisingly, according to our findings, the HPX level is decreased during OC development, while CRP and SAA1 levels are increased. Despite the fact that HPX is recycled back into the circulatory system, its level is reduced in some diseases. Thus, there is insufficient level of heme-HPX complexes, which lead to toxic activity of free heme [49]. Altered expression of HPX was found in serum from patients with OC as compared with BOT, during glycoproteomics analysis [50]. Moreover, it was down-regulated in a rat model of serous OC [32]. HPX was also decreased in serum of patients with lung cancer [51]. Interestingly, the concentrations of HPX in OC ascites were higher than normal concentrations in human plasma and serum [52]. Furthermore, HPX was overexpressed during other malignancies, like breast cancer [53] or colorectal cancer [54].

In the ELISA-based validation alterations in the levels of: APOC2, APOC4, SERPINA1, SERPINA10, and ORM1 were not confirmed. This may be the result of using set of samples that was collected externally. Moreover, there was no ELISA kit available to measure the serum concentrations of FGA. However, several of these proteins were earlier identified as potential OC markers. Concentrations of ORM1 and SERPINA1 were altered in OC patients based on serum protein glycosylation profiles [55]. Alterations in SERPINA1 expression in serum were also found when comparing OC and BOT by SELDI-TOF profiling with ELISA validation [56] and other immunoassays [57]. Under-expression of APOC2 was found in a rat model during the early and advanced stage of OC [32]. Moreover, APOC2 was proposed as a serum biomarker of other cancers [58,59]. Recently, fibrinogen has been proposed as a strong predictor for OC [60]. Therefore, it may be suggested that identified proteins are not altered in this specific set of samples that was used for validation or the alterations in their expression are subtle and do not reach statistical significance. Further studies should be conducted on a larger group of samples and for the specific histopathological types of OC.

## 4. Materials and Methods

### 4.1. Samples

Serum samples were collected from female patients operated in Clinical Hospital of Poznan University of Medical Sciences, Poland, between August 2014 and December 2015. Patients with other malignancies, OC other than epithelial or after cancer treatment, were excluded. 152 blood samples were collected on the day before surgery and incubated at room temperature for 30 min. After centrifugation for 15 min at 4000 rpm, serum samples were transferred into clean tubes and stored at −80 °C until use. 72 samples were randomly selected as a discovery set for iTRAQ-based analysis, and 80 samples were selected as a validation set for ELISA-based analysis. Study group characterization is presented in Tables S2 and S3. For iTRAQ experiment serum samples were divided into four groups containing 24 samples: OC, BOT + HC, HC, BOT. BOT + HC group consisted of 12 BOT samples and 12 HC samples that were randomly selected from the analyzed groups. ANOVA test confirmed that groups were matched in terms of age. The samples from the same group were pooled into one representative sample. Each pooled sample was prepared in quadruplicate. Sample pooling was applied to reduce individual sample variations and to increase the efficiency of the analysis and the overall number of examined samples [9,61]. The duplicates of each group were analyzed within one iTRAQ-8plex experiment. The iTRAQ-8plex analysis was conducted in duplicate. For ELISA analysis, 80 samples were used: HC (*n* = 26), OC (*n* = 23), BOT (*n* = 26), and borderline tumors (*n* = 5). To minimize systematic errors, samples were analyzed in a random order and the disease status of the patients was blinded. The study protocol was approved by Bioethical Commission of the Poznan University of Medical Sciences, Poland, (Decision No. 165/16, 04.02.2016) and the study was conducted in compliance with the Declaration of Helsinki. All of the participants signed an informed consent prior to sample collection.

### 4.2. Protein Digestion

The protein concentrations of pooled samples were determined by Bradford assay using Bradford Reagent (Merck, Darmstadt, Germany) and Protein standard—albumin from bovine serum (Merck, Darmstadt, Germany). The measurement of absorbance at 595 nm was conducted using Infinite M200PRO Microplate Reader (Tecan, Männedorf, Switzerland) and Magellan 7.1 software (Tecan, Männedorf, Switzerland). 75 µg protein aliquots of each pooled sample were cleaned up by acetone precipitation, according to iTRAQ manufacturer’s recommendations (Sciex, Framingham, MA, USA). Six volumes of cold acetone (−20 °C) were added to the cold samples (−4 °C), mixed, and incubated at −20 °C for 4 h. Pellets were further dissolved in 20 µL of the Dissolution Buffer. Samples were digested, according to the general protocol recommended by the manufacturer (Sciex, Framingham, MA, USA). Briefly, Denaturant and Reducing reagents were added to each sample tube and incubated at 60 °C for 1 h. Subsequently, Cysteine Blocking reagent was added and incubated at room temperature for 10 min. Following this, trypsin solution was added. Samples were digested overnight at 37 °C.

### 4.3. iTRAQ Labeling

After digestion, samples were labeled for 2 h with iTRAQ 8-plex tags (Sciex, Framingham, MA, USA) (dissolved in isopropanol), as follows: OC − tags 113 and 114, BOT + HC − tags 115 and 116, BOT − tags 117 and 118, HC − tags 119 and 121. Subsequently, all of the iTRAQ-labeled samples were combined into one tube. After pooling, the samples were purified using strong cation exchange (SCX) cartridge (Sciex, Framingham, MA, USA) and eluted with 10 mM KH_2_PO_4_ in 25% acetonitrile/350 mM KCl at pH 3.0.

### 4.4. nanoLC-MALDI-TOF/TOF MS/MS Analysis

The obtained iTRAQ-labeled peptides were further subjected to nano-LC separation using Easy-nano LC II (Bruker Daltonics, Bremen, Germany), coupled to a fraction collector—Proteineer-fc II (Bruker Daltonics, Bremen, Germany). The nanoflow HPLC set consisted of a trap column (NS-MP-10 BioSphere C18, NanoSeparations, Nieuwkoop, The Netherlands) (length 20 mm, I.D. 100 μm, particle size 5 μm, pore size 120 Å) and C18 separation column (PepMap 100, Thermo Scientific, Waltham, MA, USA) (length 150 mm, I.D. 75 μm, particle size 3 μm, pore size 100 Å). The separation was conducted with a flow rate of 300 nL/min and the following mobile phases: A. 0.1% trifluoroacetic acid (TFA) in water, B. 0.1% TFA in acetonitrile. A linear gradient was from 2 to 50% of phase B for 114 min. 18 min after the initiation of the analytical gradient, the fractions were automatically mixed with a MALDI matrix solution prepared by mixing: 36 μL of α-cyano-4-hydroxycinnamic acid (HCCA) saturated solution of 0.1% TFA in water and acetonitrile (10:90 *v*/*v*), 784 μL of 0.1% TFA and acetonitrile (5:95 *v*/*v*), 8 μL of 10% TFA, and 8 μL of 100 mM NH_4_H_2_PO_4_. 80 nL of each eluent mixed with 420 nL of the matrix was automatically spotted onto the MALDI target plate (MTP AnchorChip 384, Bruker Daltonics, Bremen, Germany). In total, 384 fractions were obtained. The nanoLC system coupled to a fraction collector was controlled by HyStar version 3.2 software (Bruker Daltonics, Bremen, Germany). Subsequently, fractions were analyzed using MALDI-TOF/TOF (UltrafleXtreme, Bruker Daltonics, Bremen, Germany) instrument in the positive ion mode. Peptide Calibration Standard mixture (Bruker Daltonics, Bremen, Germany) was used to externally calibrate MS spectra. The spectra acquisition, data processing, and evaluation were performed by FlexControl 3.4 and FlexAnalysis 3.4. software (Bruker Daltonics, Bremen, Germany). 5000 laser shots were accumulated to acquire each spectrum in MS and MS/MS modes. A list of precursor peptides was selected for MS/MS analysis using WARP-LC version 1.3 software (Bruker Daltonics, Bremen, Germany) after screening all of the spots in MS-positive reflectron mode in the mass range of 700–3500 Da. Three different signal-to-noise thresholds were set: 15, 10, and 5. Each analysis with specific signal-to-noise threshold was performed in triplicate with the use of exclusion scheduled precursor list created in ProteinScape version 3.0 software (Bruker Daltonics, Bremen, Germany). Fragmentation of the precursor ions was performed using collision induced dissociation and argon as the collision gas.

### 4.5. Database Search and Data Processing

The obtained MS/MS data were further processed using ProteinScape version 3.0 software (Bruker Daltonics, Bremen, Germany), SwissProt database and Mascot version 2.4.1 search engine (Matrix Science, London, UK). The general protein search parameters were, as follows: trypsin digestion, 1 missed cleavages, peptide mass tolerance: 30 ppm; MS/MS fragment mass tolerance: 0.7 Da; peptide charge: 1+; monoisotopic mass; methylthio (C) as fixed modification; oxidation (M), iTRAQ8plex (K, N-term, Y) as variable modifications. The false discovery rate was kept below 1% for all the peptide identifications based on decoy counts. All of the results obtained from analysis with different signal-to-noise thresholds and two iTRAQ experiments were compiled into one list generated by the ProteinScape version 3.0 software (Bruker Daltonics, Bremen, Germany). Quantitative analysis was automatically performed in WARP-LC version 1.3 software (Bruker Daltonics, Bremen, Germany). The median iTRAQ reporter ions peak area ratios of labeled peptides from the MS/MS spectra were calculated. The following median ratios were included: 113/115, 113/116, 114/115, 114/116, 113/117, 113/118, 114/117, 114/118, 113/119, 113/121, 114/119, 114/121, 117/119, 117/121, 118/119, 118/121. The median values for all the peptides for each label-tag were adjusted to normalize the obtained results. Subsequently, data were exported into Excel (Microsoft, Redmond, WA, USA). Finally, the average values from ratios corresponding to comparisons of different groups (OC, BOT + HC, HC, BOT) were calculated. The ratios above 1.5 and below 0.66 were considered to be statistically significant. Identified proteins were further analyzed using PANTHER (protein annotation through evolutionary relationship) Classification System database (http://pantherdb.org/) [10] and Human Proteome Map portal (HPM) (http://www.humanproteomemap.org) [62]. Interaction networks between differently expressed proteins were analyzed by STRING (https://string-db.org/).

### 4.6. Quantification of Individual Proteins by ELISA

Commercial ELISA kits (Cloud-Clone Corp., Houston, TX, USA) were used to quantify levels of selected proteins: Alpha-1-antitrypsin, Alpha-1-acid glycoprotein 1, C-reactive protein, Hemopexin, Serotransferrin, Serum albumin, Serum amyloid A protein, Apolipoprotein C-II, Apolipoprotein C-IV, and Protein Z-dependent protease inhibitor. The analyses were performed, according to the manufacturer’s instructions, using Infinite M200PRO Microplate Reader (Tecan, Männedorf, Switzerland) and Magellan 7.1 software (Tecan, Männedorf, Switzerland). A new set of 80 samples was used to determine the concentrations of the proteins: OC (*n* = 23), borderline tumors (*n* = 5), HC (*n* = 26), and BOT (*n* = 26). Additionally, two OC markers: CA125 and HE4 were quantified by electrochemiluminescence immunoassay on Roche Cobas System (Roche Diagnostics, Indianapolis, IN, USA) for OC and BOT, as part of a standard procedure for adnexal mass assessment in the Central Hospital Laboratory. Statistical analysis of the obtained results was performed using MetaboAnalyst 4.0. (http://www.metaboanalyst.ca/). The utility of determined proteins was calculated for the following group comparisons: OC vs. HC, OC vs. BOT, OC vs. HC + BOT, BOT vs. HC. Moreover, an additional analysis comparing the borderline group with OC and BOT was conducted. P-values based on *t*-test were calculated and the results <0.05 were considered to be statistically significant. Differences in protein expression were also analyzed using univariate and multivariate receiver operating characteristic (ROC) curve. Additionally, in order to compare the utility of the studied proteins to differentiate between OC stages, we divided OC into two groups: stages I + II (*n* = 8) and stages III + IV (*n* = 14). Then, ANOVA analysis was performed to compare different stages of OC with BOT and HC.

## 5. Conclusions

To sum up, the applied approach based on iTRAQ technology coupled with mass spectrometry showed great potential for serum proteomic studies. Several proteins that are differently expressed in OC patients were identified, which shed light on the molecular signaling pathways associated with OC development. Subsequently, these findings were validated in external set of samples, using the quantitative method—ELISA. TF, SAA1, HPX, CRP, and ALB were identified as potential OC biomarkers. Our results confirmed that identified proteins show the capacity to become markers of carcinogenesis. Due to the significant role of the TF, SAA1, HPX, CRP, and ALB in OC development, these proteins should be further examined in samples that are derived from an early stage of OC. Our research supply evidence that proposed proteins may be used in a multimarker test and have utility as a novel screening tool to detect OC.

## Figures and Tables

**Table 1:** ITRAQ ratios and protein class (according to protein annotation through evolutionary relationship (PANTHER) database) of the differently expressed proteins.

Accession	Protein Name	Protein Class	iTRAQ Ratios			
			OC/HC + BOT	OC/HC	OC/BOT	BOT/HC
ORM1	α-1-acid glycoprotein 1	-	0.56	0.81	0.64	1.28
SERPINA1	α-1-antitrypsin	Enzyme modulator	0.75	0.48	0.89	0.54
APOC2	Apolipoprotein C-II	-	1.24	0.96	1.67	0.59
APOC4	Apolipoprotein C-IV	-	1.04	0.74	1.28	0.59
CRP	C-reactive protein	-	2.47	2.37	3.07	0.78
FGA	Fibrinogen alpha chain	-	2.31	1.97	1.83	1.09
HPX	Hemopexin	Hydrolase	0.44	0.97	0.67	1.55
SERPINA10	Protein Z-dependent protease inhibitor	Enzyme modulator	0.98	0.76	1.20	0.60
TF	Serotransferrin	Transfer/carrier protein; Receptor; Hydrolase; Defense/immunity protein	0.64	0.91	0.65	1.39
ALB	Serum albumin	Transfer/carrier protein	0.60	0.75	0.58	1.33
SAA1	Serum amyloid A protein	Transporter; Transfer/carrier protein; Defense/immunity protein	3.35	3.59	4.33	0.83

iTRAQ isobaric Tags for Relative and Absolute Quantification; OC ovarian cancer; BOT benign ovarian tumor; HC healthy controls.

**Table 2:** ELISA data-based statistical results: *p*-values and AUC in discriminating: ovarian cancer (OC) vs. healthy controls (HC) + benign ovarian tumors (BOT), OC vs. HC, OC vs. BOT, BOT vs. HC. * CA125 and HE4 were only quantified in OC and BOT groups as standard hospital procedure.

Protein	OC vs. HC + BOT		OC vs. HC		OC vs. BOT		BOT vs. HC	
	AUC	*p*-Values	AUC	*p*-Values	AUC	*p*-Values	AUC	*p*-Values
ORM1	0.518	0.4022	0.505	0.5679	0.540	0.4580	0.536	0.6967
SERPINA1	0.622	0.0949	0.616	0.1091	0.627	0.2083	0.515	0.7274
APOC2	0.621	0.1187	0.635	0.1377	0.607	0.2330	0.523	0.6806
APOC4	0.595	0.256	0.620	0.1769	0.569	0.5362	0.561	0.4416
CRP	0.777	<0.0001	0.761	<0.0001	0.793	<0.0001	0.544	0.3409
HPX	0.779	<0.0001	0.762	0.0011	0.796	<0.0001	0.540	0.4167
SERPINA10	0.602	0.2756	0.559	0.5114	0.645	0.1834	0.580	0.6550
TF	0.851	<0.0001	0.845	<0.0001	0.858	<0.0001	0.531	0.6127
ALB	0.787	<0.0001	0.758	<0.0001	0.875	<0.0001	0.596	0.3449
SAA1	0.825	<0.0001	0.799	<0.0001	0.851	<0.0001	0.593	0.1719
CA125 *	-	-	-	-	0.965	<0.0001	-	-
HE4 *	-	-	-	-	1.0	<0.0001	-	-

ORM1 Alpha-1-acid glycoprotein 1; SERPINA1 Alpha-1-antitrypsin; APOC2 Apolipoprotein C-II; APOC4 Apolipoprotein C-IV; CRP C-reactive protein; HPX Hemopexin; SERPINA10 Protein Z-dependent protease inhibitor; TF Serotransferrin; ALB Albumin; SAA1 Serum amyloid A1; CA125 Cancer Antigen 125; HE4 human epididymis protein 4.

## Supplementary Materials

Supplementary materials can be found at http://www.mdpi.com/1422-0067/19/8/2240/s1.

## Abbreviations

OC	ovarian cancer
FIGO	International Federation of Gynaecology and Obstetrics
CA125	cancer antigen 125
HE4	human epididymis protein 4
RMI	Risk of Malignancy Index
SILAC	stable isotope labelling by amino acids in cell culture
ICAT	Isotope-coded affinity tag
ITRAQ	isobaric Tags for Relative and Absolute Quantification
MALDI-TOF	matrix-assisted laser desorption/ionization time-of-flight
MS	mass spectrometry
BOT	benign ovarian tumors
HC	Healthy controls
ELISA	enzyme-linked immunosorbent assay
SC	sequences coverage
MW	molecular weight
CRP	C-reactive protein
FGA	Fibrinogen alpha chain
APOC2	Apolipoprotein C-II
SAA1	Serum amyloid A1
ORM1	Alpha-1-acid glycoprotein 1
SERPINA1	Alpha-1-antitrypsin
HPX	Hemopexin
TF	Serotransferrin
ALB	Serum albumin
CV	Coefficient of variation
SCX	strong cation exchange
LC	liquid chromatography
TFA	trifluoroacetic acid
HCCA	α-cyano-4-hydroxycinnamic acid
HPM	Human Proteome Map
ROC	receiver operating characteristic curve
