# Supplementary material for: Understanding Ovarian Cancer: iTRAQ-Based Proteomics for Biomarker Discovery
Source: Int J Mol Sci. 2018 Jul 31;19(8):2240. doi: 10.3390/ijms19082240 (PMC6121953; doi:10.3390/ijms19082240)
Supplement: Supplementary file 1 [file ijms-19-02240-s001.zip › Table S2-S5.docx]

Supplementary

Understanding Ovarian Cancer: iTRAQ-Based Proteomics for Biomarker Discovery

Agata Swiatly ^1^, Agnieszka Horala ^2^, Jan Matysiak ^1^, Joanna Hajduk ^1^, Ewa Nowak-Markwitz ^2^ and Zenon J. Kokot ^1,^*

^1^ Department of Inorganic and Analytical Chemistry, Poznan University of Medical Sciences, ul. Grunwaldzka 6, 60-780 Poznań, Poland; agataswiatly@gmail.com (A.S.); jmatysiak@ump.edu.pl (J.M.); jo.hajduk@gmail.com (J.H.)

^2^ Gynecologic Oncology Department, Poznan University of Medical Sciences, ul. Polna 33, 60-535 Poznań, Poland; agnieszka0lemanska@gmail.com (A.H.); ewamarkwitz@poczta.fm (E.N.-M.)

***** Correspondence zkokot@ump.edu.pl; Tel.: +48-61-854-6610

**Table S2.** Characterization of the study groups.

|  | **Number of samples** | | **Median Age**  **(Range)** | | **Median BMI (Range)** | | **% of Postmenopausal** | |
| --- | --- | --- | --- | --- | --- | --- | --- | --- |
|  | **iTRAQ analysis** | **ELISA validation** | **iTRAQ analysis** | **ELISA validation** | **iTRAQ analysis** | **ELISA validation** | **iTRAQ analysis** | **ELISA validation** |
| **HC** | 24 | 26 | 59  (33-69) | 68  (19-63) | 25.64  (21-40) | 25.78  (18-34) | 75% | 46% |
| **BOT** | 24 | 26 | 51  (38-72) | 32  (17-66) | 25.87  (21-39) | 23.82  (17-33) | 50% | 10% |
| **OC** | 24 | 23 | 57  (32-72) | 59  (46-76) | 25.64  (20-36) | 26.42  (18-38) | 75% | 78% |
| **Borderline tumor** | - | 5 | - | 45  (37-52) | - | 27.69  (17-31) | - | 20% |

**Table S3.** Characterization of OC group.

| **Histopathological Type** | **Number of samples** | | **Percentage (%)** | |
| --- | --- | --- | --- | --- |
|  | **iTRAQ analysis** | **ELISA validation** | **iTRAQ analysis** | **ELISA validation** |
| Serous | 10 | 5 | 42 | 24 |
| Endometrioid | 4 | 1 | 17 | 5 |
| Mucinous | 1 | 1 | 4 | 5 |
| Clear cell | 2 | 3 | 8 | 14 |
| Undifferentiated | 4 | 8 | 17 | 38 |
| Non identified | 3 | 3 | 12 | 14 |
| **FIGO stage** | | | | |
| I | 5 | 6 | 21 | 27 |
| II | 1 | 2 | 4 | 9 |
| III | 17 | 14 | 71 | 64 |
| IV | 1 | 0 | 4 | 0 |

**Table S4.** ELISA data-based statistical results: p-values and AUC in discriminating: borderline tumors vs OC and borderline tumors vs BOT.

| Protein | Bordeline tumours vs OC | | Borderline tumours vs BOT | |
| --- | --- | --- | --- | --- |
|  | AUC | p-values | AUC | p-values |
| TF | 0.878 | 0.0093 | 0.585 | 0.4914 |
| SAA1 | 0.782 | 0.0369 | 0.577 | 0.3872 |
| HPX | 0.904 | 0.0023 | 0.642 | 0.4895 |
| CRP | 0.626 | 0.3847 | 0.765 | 0.0335 |
| ALB | 0.852 | 0.0110 | 0.638 | 0.5372 |
| APOC2 | 0.782 | 0.0938 | 0.723 | 0.2477 |
| APOC4 | 0.543 | 0.8310 | 0.638 | 0.5571 |
| SERPINA1 | 0.739 | 0.3104 | 0.546 | 0.7709 |
| SERPINA10 | 0.748 | 0.0851 | 0.561 | 0.1520 |
| ORM1 | 0.513 | 0.7658 | 0.507 | 0.8915 |
| HE4 | 0.930 | 0.1302 | 0.700 | 0.0024 |
| CA125 | 0.930 | 0.0791 | 0.562 | 0.8864 |

**Table S5.** Results of ANOVA test (p-value, false discovery rate – FDR and post-hoc tests), which present the ability of CRP, ALB, SAA1, TF and HPX to differentiate the following groups of samples: FIGO stages I + II, FIGO stages III – IV, HC, BOT.

| **Protein** | **p-value** | **FDR** | **Post-hoc tests** |
| --- | --- | --- | --- |
| C-reactive protein | <0.001 | <0.001 | I+II – III-IV  III + IV – HC  III + IV - BOT |
| Albumin | <0.001 | <0.001 | I+II – HC  I + II – BOT  I + II – III + IV  III + IV – HC  III + IV - BOT |
| Amyloid A1 | <0.001 | <0.001 | I+II – HC  I + II – BOT  III + IV – HC  III + IV - BOT |
| Serotransferrin | <0.001 | <0.001 | I+II – HC  I + II – BOT  III + IV – HC  III + IV - BOT |
| Hemopexin | <0.001 | <0.001 | III + IV – HC  III + IV - BOT |
